# Ward-Based Noninvasive Ventilation for Acute Hypercapnic Respiratory Failure Unrelated to Chronic Obstructive Pulmonary Disease

**DOI:** 10.1155/2021/4835536

**Published:** 2021-12-21

**Authors:** Bandar M. Faqihi, Dhruv Parekh, Samuel P. Trethewey, Julien Morlet, Rahul Mukherjee, Alice M. Turner

**Affiliations:** ^1^Institute of Applied Health Research, University of Birmingham, Birmingham, UK; ^2^Respiratory Therapy Department, College of Applied Medical Sciences, King Saud Bin Abdul Aziz University for Health Sciences, Saudi Arabia; ^3^University Hospitals Birmingham NHS Foundation Trust, Birmingham, UK; ^4^Birmingham Acute Care Research Group, Institute of Inflammation and Ageing, University of Birmingham, Birmingham, UK

## Abstract

**Background:**

The use of ward-based noninvasive ventilation (NIV) for acute hypercapnic respiratory failure (AHRF) unrelated to chronic obstructive pulmonary disease (COPD) remains controversial. This study evaluated the outcomes and failure rates associated with NIV application in the ward-based setting for patients with AHRF unrelated to COPD.

**Methods:**

A multicentre, retrospective cohort study of patients with AHRF unrelated to COPD was conducted. COPD was not the main reason for hospital admission, treated with ward-based NIV between February 2004 and December 2018. All AHRF patients were eligible; exclusion criteria comprised COPD patients, age < 18 years, pre-NIV pH < 7.35, or a lack of pre-NIV blood gas. In-hospital mortality was the primary outcome; univariable and multivariable models were constructed. The obesity-related AHRF group included patients with AHRF due to obesity hypoventilation syndrome (OHS), and the non-obesity-related AHRF group included patients with AHRF due to pneumonia, bronchiectasis, neuromuscular disease, or fluid overload.

**Results:**

In total, 479 patients were included in the analysis; 80.2% of patients survived to hospital discharge. Obesity-related AHRF was the indication for NIV in 39.2% of all episodes and was the aetiology with the highest rate of survival to hospital discharge (93.1%). In the multivariable analysis, factors associated with a higher risk of in-hospital mortality were increased age (odds ratio, 95% CI: 1.034, 1.017–1.051, *P* < 0.001) and pneumonia on admission (5.313, 2.326–12.131, *P* < 0.001). In the obesity-related AHRF group, pre-NIV pH < 7.15 was associated with significantly increased in-hospital mortality (7.800, 1.843–33.013, *P*=0.005); however, a pre-NIV pH 7.15–7.25 was not associated with increased in-hospital mortality (2.035, 0.523–7.915, *P*=0.305).

**Conclusion:**

Pre-NIV pH and age have been identified as important predictors of surviving ward-based NIV treatment. Moreover, these data support the use of NIV in ward-based settings for obesity-related AHRF patients with pre-NIV pH thresholds down to 7.15. However, future controlled trials are required to confirm the effectiveness of NIV use outside critical care settings for obesity-related AHRF.

## 1. Introduction

NIV has been widely used in intensive care units (ICUs) for many years to treat conditions such as acute exacerbations of COPD (AECOPD) and is regarded as effective for avoidance of endotracheal intubation [1] and decreasing mortality in patients with AHRF. In 2000, findings from a randomised controlled trial supported the use of ward-based NIV for patients with acute exacerbation of COPD outside ICUs (in general medical wards) as it improved the mortality rate and reduced the need for invasive mechanical ventilation [2]. Currently, managing patients who require NIV in an ICU setting is resource-intensive and in the current economic climate, where healthcare budgets are increasingly limited, maximizing cost-effectiveness by enhancing ward-based care is important. However, the use of ward-based NIV for AHRF unrelated to COPD is not widely established, perhaps due to concerns over its efficacy and safety.

In addition, poor understanding of the role of ward-based care for AHRF unrelated to COPD limits a hospital's ability to design care pathways. In COPD, findings from a randomised controlled trial “YONIV trial” (Yorkshire Non-Invasive Ventilation trial) done in 2000 supported the use of ward-based NIV for patients with AECOPD outside critical care settings (in wards-based settings) as it improved the mortality rate and reduced the need for invasive mechanical ventilation [2]. Since the trial, numerous studies have supported the use of NIV outside of the critical care environment for the AECOPD group with AHRF [3–13]. In addition, using prospectively collected data for COPD patients with AHRF who underwent NIV from 2004 to 2009 at a single centre in the UK, NIV in ward-based settings was an effective treatment in hospitalised AECOPD patients with severe AHRF [14].

With regards to the conditions other than COPD, the use of ward-based NIV is excluded or limited in many hospitals' pathways based on unclear and limited evidence regarding their prognosis or utility of treatment outside critical care settings. Over time, NIV was used for some clinical conditions such as restrictive lung diseases and OHS, and most of the data arise from cohort studies [15–17]. Therefore, the purpose of this study was to evaluate outcomes and failure rates associated with NIV application in the ward-based environment for patients with AHRF unrelated to COPD. We further chose to analyse efficacy based on pre-NIV pH thresholds, as we did previously in COPD, to inform care pathway design.

## 2. Methods

This was a multicentre, retrospective cohort study of patients with AHRF unrelated to COPD treated by NIV in ward-based settings. Data were collected prospectively as part of service evaluation and were analysed retrospectively. This study was conducted in three hospitals in Birmingham, United Kingdom: Queen Elizabeth Hospital Birmingham (QEHB), Birmingham Heartlands Hospital (BHH), and Good Hope Hospital (GHH). In total, 479 subjects were enrolled using the NIV databases of the three hospitals between February 2004 and December 2018, including all patients receiving ward-based NIV where the cause of AHRF was not COPD. The study was approved by both clinical and research departments within the hospital as being a study of routinely collected data for which no external approvals were required, beyond the local ones which they granted. Data from the COPD patients in the cohort have been published previously [18].

### 2.1. Inclusion and Exclusion Criteria

All adult patients treated with ward-based NIV for AHRF unrelated to COPD at the time of admission were enrolled in this study. All patients enrolled had a pre-NIV pH < 7.35 and pCO_2_ > 6.0 kPa. The exclusion criteria were as follows: COPD patients (primary diagnosis or with previous or new clinical diagnosis of COPD), patients <18 years old, pre-NIV pH > 7.35, or a lack of pre-NIV blood gas.

### 2.2. Analysis

The primary outcome was in-hospital mortality, death occurring during the hospital stay. Factors associated with in-hospital mortality (e.g., pre-NIV pH, age, gender, and disease) were assessed initially using univariable analysis and then using a multivariable model to determine independent associations. Subgroup analyses according to the different conditions necessitating the use of NIV in ward-based care settings were also completed. Secondary outcomes were NIV failure, prognosis to intubation, NIV duration (days), and time from diagnosis to NIV application (minutes). During obtaining the database, there were missing data for some secondary outcomes. These missing data were extracted with the help from the healthcare providers by the hospitals' medical records such that missing elements were <1% of cases after medical record review.

The statistical analyses were performed using IBM SPSS Statistics Version 24. Statistical significance was taken as a *p* value <0.05. The Kolmogorov–Smirnov test and visual assessment were used to determine data normality. Data including pre-NIV pH, age, NIV duration, and time from diagnosis to NIV application were nonparametric and therefore expressed as median (interquartile range (IQR)) and compared between outcome groups by using the Mann–Whitney and Kruskal–Wallis tests. Number (percentage) was used to present categorical data, such as aetiology of AHRF, and the Chi-Square test was used for analyses of associations between categorical variables and outcomes. Multivariable analysis was then performed using binary logistic regression (backward stepwise Wald) if the factors were significant in a univariable analysis to identify independent predictors of in-hospital mortality. Similar approaches were used to assess the relationship between clinical characteristics and secondary outcomes.

## 3. Results

### 3.1. Patient Characteristics

In total, 479 patients were included in the study. Patients' baseline characteristics split by diagnostic conditions, obesity-related AHRF, and non-obesity-related AHRF are summarised in Table 1. The characteristics of obesity-related AHRF and non-obesity-related AHRF are shown in Supplementary Tables 1 and 2. Overall, almost 20% of the included patients died in the hospital. Patients were further subdivided into six groups based on their underlying conditions: obesity-related AHRF, which is defined as the combination of daytime alveolar hypoventilation, obesity (BMI <30 kg/m^2^), and sleep-disordered breathing, pneumonia, bronchiectasis, neuromuscular disease, fluid overload (included pulmonary oedema, heart failure, and metabolic/renal failure), and others (e.g., asthma, postoperative RF) (Table 2). Obesity-related AHRF had the lowest in-hospital mortality rate compared to other diagnosis conditions.

In the univariable analysis for the two subgroups (obesity- and non-obesity-related AHRF), the patients who died in-hospital were older than those who survived to discharge. There were statistically significant differences in survival between the three hospitals (*P*=0.017), as well as significant differences between survivors and those who died with regards to the underlying diagnosis. Pre-NIV pH was higher in the survived to discharge group compared to the in-hospital mortality group. More than two-thirds of the in-hospital mortality group had NIV failure. In one of the included hospitals (BHH, *n* = 237), more data were available, which enabled additional analyses in this subgroup. There were significant differences between the groups (survived vs. died) in the number of days using NIV and the proportion of patients who were treated with domiciliary NIV. No significant differences were noted between the two groups in the time from diagnosis to NIV application (Supplementary Tables 1 and 2).

**Figure 1:**
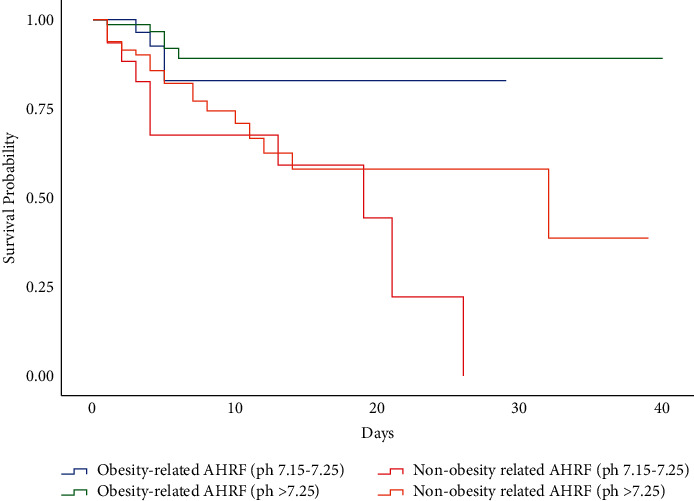
Kaplan–Meier curves illustrating in-hospital mortality in patients stratified by diagnostic condition (obesity-related AHRF vs. non-obesity-related AHRF) (log-rank: *p*=0.001).

In multivariable logistic regression, in total non-COPD AHRF group, significant predictors of in-hospital mortality were pre-NIV pH < 7.25, age, and underlying cause of AHRF, whereby the aetiology with the best prognosis was obesity-related AHRF (Table 3). This condition was taken as the reference value, and hazard ratios for death relative to this were calculated in the regression models. However, in the obesity-related AHRF subgroup, pre-NIV pH of below 7.15 was associated with a significant increase in mortality (7.800, *P*=0.005) but not between 7.15 and 7.25 (2.035, *P*=0.305). Moreover, age was not associated with a significant increase in mortality (1.030, *P*=0.231) when compared to the age in the non-obesity-related AHRF subgroup. In the BHH subgroup, where additional data were available, we were also able to assess the contribution of domiciliary NIV to the model; receipt of this appeared to be protective. A Kaplan–Meier curve was constructed comparing in-hospital mortality in groups split by aetiology of AHRF (obesity-related AHRF vs. non-obesity-related AHRF) (Figure 1).

## 4. Discussion

Our study has shown that patients with obesity-related AHRF have a high rate of survival to hospital discharge (93.6%) when managed in ward-based settings. We have also shown that obesity-related AHRF patients with a pre-NIV pH between 7.15 and 7.25 exhibit similar prognosis to patients with a higher pre-NIV pH, unlike in other conditions causing AHRF. This suggests that obesity-related AHRF can be safely managed outside the ICU at pre-NIV pH levels down to 7.15. However, in other conditions reported in our paper, patients with a pre-NIV pH < 7.25 might require management in the ICU setting due to their poor prognosis. The exception to this would be if there was a ceiling of treatment set, whereby it was decided that escalation beyond the ward environment was inappropriate.

### 4.1. Survival Rates When Managed with Ward-Based NIV

Overall, 80.2% of patients survived to hospital discharge, which is greater than the survival rate reported in non-COPD AHRF patients by Carter et al. and in the UK national audit of AHRF patients who had COPD (79.55% and 75%, respectively) [19, 20]. This suggests that hospitals could consider modifying their NIV care pathways to accept more non-COPD AHRF cases into ward-based settings, as their prognosis is equal or better than those already routinely managed in this setting. Whilst rates of in-hospital mortality in recent studies appear higher than the landmark randomised controlled trial in COPD [2], this may be related to the selection of patients who were not expected to survive, either due to their condition and its severity or due to comorbid disease [21].

The in-hospital mortality rate of 6.9% for patients with obesity-related AHRF was significantly lower than for other conditions which strongly suggests that NIV should be considered for use in obesity-related AHRF in ward-based settings, given that their risk of a poor outcome is lower. This finding is consistent with the results of a study of patients managed in a critical care setting, in which mortality was found to be lower in obesity-related respiratory failure patients compared to COPD patients managed with NIV [17]. Moreover, this is consistent with a recent study that reported a 15% mortality rate for the obesity-related respiratory failure group which was lower compared to the other diagnostic groups [20].

Patients with pneumonia had the highest rate of in-hospital mortality (32.1%), with a high odds ratio of mortality when compared to obesity-related AHRF patients (5.313, *P* < 0.0001) and other diagnostic groups. This finding is in line with the National Confidential Enquiry into Patient Outcome and Death (NCEPOD) data, which indicated that patients with pneumonia experienced higher in-hospital mortality [21]. Moreover, the British Thoracic Society/Intensive Care Society guidelines for the ventilatory management of acute hypercapnic respiratory failure in adults already recommend against the use of ward‐based NIV in patients with pneumonia [22]. The higher in-hospital mortality rate noticed in patients with pneumonia highlights and emphasises the importance of the NCEPOD recommendation that “Early senior review and escalation planning is essential to ensure these patients receive appropriate treatment in the correct location.” [21].

This study showed that a significantly higher proportion of the in-hospital mortality group had NIV failure compared to the discharged group (83.2% vs. 5.7%, *P* < 0.0001). One possible explanation for this is that most of them may have received NIV as a ceiling treatment, which is not fully reported in the database; this might explain why they were being treated in ward-based setting, instead of critical care settings with advanced treatment.

### 4.2. Predictors of In-Hospital Mortality

Pre-NIV pH was a significant predictor of in-hospital mortality when it was analysed as a continuous variable and when it was grouped by thresholds. The importance of pre-NIV pH seen in our study was consistent with the findings of the UK national audit of COPD patients treated with NIV [19] where pre-NIV pH < 7.15 and 7.15–7.25 were associated with a higher risk of mortality (2.223, *P*=0.021, and 1.865, *P*=0.023, respectively). However, in contrast to the findings seen in COPD patients in the national audit, we were able to show that there was no difference in the rate of in-hospital mortality between obesity-related AHRF patients with pre-NIV pH 7.15–7.25 and those with pre-NIV pH < 7.25 and that this effect was driven by low death rates in the obesity subgroup at lower pH levels. This suggests that a pre-NIV pH threshold of 7.15–7.25 could be chosen for managing obesity-related AHRF patients in lower intensity settings (outside the ICU). We have previously shown this in COPD patients also [14], but the message appeared stronger in the obesity-related AHRF subgroup here than in our prior data.

Age was also an important predictor of in-hospital mortality in non-COPD AHRF patients treated with NIV. This is consistent with studies done on AHRF due to COPD [14, 23] and AHRF unrelated to COPD [20]. It was expected that an association between age and mortality would be found since age is not necessarily a limitation to the treatment and, in general, older age is associated with worse prognosis.

The time from AHRF to NIV application was no different in the in-hospital mortality group. The British Thoracic Society ‘Quality Standards for Acute NIV in Adults' notes that “Patients who meet evidence‐based criteria for acute NIV should start NIV within 60 minutes of the blood gas result associated with the clinical decision to provide NIV and within 120 minutes of hospital arrival for patients who present acutely” [24]. This is because delays in treatment have been associated with reduced survival; however, it is also notable that some patients with COPD deteriorate late, and these also represent a poor prognostic group [23]. A longer wait for NIV application could result in high numbers of emergency hospital admissions, poor NIV capacity, or inadequate clarity within the hospital's NIV pathway. Notably, the time from diagnosis to NIV application was generally at or close to the national standard of 120 mins (120 vs. 140 minutes, survived vs. died) in our group which may have reduced power to detect differences based on this factor.

### 4.3. Strengths and Limitations

The key strengths of this study include the multiple centres with a large cohort size, which is larger than other recent cohort studies targeting the same population [20], and the detailed data available particularly at BHH, which allowed assessment of the impact of timing of acute NIV treatment as well as the impact of previous domiciliary NIV. The study's findings, however, were limited by the uncontrolled, retrospective cohort design.

## 5. Conclusion

In summary, based on our difficulty in ascertaining from the database which patients had NIV as ceiling of treatment and what were the comorbidities, from April 2019, we have upgraded our database to capture that information in real time. We have built upon previous work within COPD patients, in which pre-NIV pH has been identified as an important predictor of surviving ward-based NIV treatment. Our findings support the use of NIV in ward-based settings for obesity-related AHRF patients with pre-NIV pH thresholds from 7.15 upwards. Based on the promising outcomes for the obesity-related AHRF group in this study and in other recent studies, future controlled trials are required to prove the effectiveness of using NIV outside critical care settings for obesity-related AHRF.

## Figures and Tables

**Table 1:** Participant baseline characteristics.

Characteristics	Median (IQR) OR *n* (%)			*P*
	Total *n* = 479	Obesity-related AHRF^*∗*^*n* = 188	Non-obesity-related AHRF *n* = 291	
Age (years)	73 [62–81]	69 [60–75]	76 [65.75–84]	<0.001
Male	192 (40.1)	66 (35.1)	126 (43.3)	<0.001
Female	287 (59.9)	122 (64.9)	165 (56.7)	0.011
Survival to discharge	384 (80.2)	175 (93.1)	209 (71.8)	0.083
In-hospital mortality	95 (19.8)	13 (6.9)	82 (28.2)	<0.001
Pre-NIV pH	7.27 [7.21–7.31]	7.27 [7.23–7.31]	7.26 [7.20–7.31]	0.042
*Pre-NIV pH thresholds*				
pH > 7.15	61 (12.7)	16 (8.5)	45 (15.5)	<0.001
pH 7.15–7.25	138 (28.8)	50 (26.6)	88 (30.2)	0.001
pH < 7.25	280 (58.5)	122 (64.9)	158 (54.3)	0.031
NIV failure	101 (21.1)	15 (8.0)	86 (29.6)	<0.001

*Subgroup (BHH*)				
Duration of NIV (days)	5 [3–9]	6 [4–10]	4 [2–9]	0.015
RF to NIV (minutes)	123 [63.5–302.5]	123 [63.5–302.5]	122 [60.0–316.0]	0.692
Domiciliary NIV	44 (23.4)	27 (14.4)	17 (5.8)	0.132

^*∗*^Obesity-related AHRF patients due to obesity hypoventilation syndrome; IQR, interquartile range; BHH, Birmingham Heartlands Hospital; NIV, noninvasive ventilation; RF, respiratory failure; AHRF: acute hypercapnic respiratory failure.

**Table 2:** Prevalence of conditions causing AHRF.

*n* (%)			
Diagnosis	Total	Survived to discharge	In-hospital mortality
Pneumonia	53 (11.1)	36 (67.9)	17 (32.1)
Bronchiectasis	40 (8.4)	30 (75)	10 (25)
Obesity-related AHRF	188 (39.2)	175 (93.1)	13 (6.9)
Neuromuscular disease	85 (17.7)	63 (74.1)	22 (25.9)
Fluid overload	48 (10)	35 (72.9)	13 (27.1)
Other	65 (13.6)	45 (69.2)	20 (30.8)
Total	479 (100)	384 (80.2)	95 (19.8)

AHRF: acute hypercapnic respiratory failure.

**Table 3:** Multivariable logistic regression demonstrating the predictors of in-hospital mortality.

Variable		Odds ratio	*P*
Total non-COPD AHRF			
Pre-NIV pH > 7.25	pH > 7.15	2.223^a^ (1.130–4.375)	0.021
	pH 7.15–7.25	1.865^a^ (1.091–3.187)	0.023
Pre-NIV pH^*∗*^	0.004 (0.000–0.062)	<0.001	
Pneumonia on admission	5.313^b^ (2.326–12.131)	<0.001	
Bronchiectasis on admission	4.236^b^ (1.680–10.679)	0.002	
NMD on admission	4.038^b^ (1.888–8.636)	0.003	
Fluid overload on admission	3.735^b^ (1.556–8.966)	0.021	
Age	1.034 (1.017–1.051)	>0.001	
Subgroup (BHH): domiciliary NIV	0.065 (0.009–0.488)	0.008	

Subgroup: obesity-related AHRF			
Pre-NIV pH > 7.25	pH > 7.15	7.800^a^ (1.843–33.013)	0.005
	pH 7.15–7.25	2.035^a^ (0.523–7.915)	0.305
Age	1.030 (0.981–1.082)	0.231	

Subgroup: non-obesity-related AHRF			
Pre-NIV pH > 7.25	pH > 7.15	4.538^a^ (1.694–9.354)	0.002
	pH 7.15–7.25	1.843^a^ (1.038–3.272)	0.037
Age	1.030 (1.010–1.050)	0.005	

^a^The odds ratio is against the reference group: pH < 7.25, ^b^The odds ratio is against the reference group: obesity-related AHRF. ^*∗*^The pre-NIV pH was analysed as the continuous variable; COPD: chronic obstructive pulmonary disease; AHRF: acute hypercapnic respiratory failure; NIV: noninvasive ventilation; NMD: neuromuscular disease; BHH: Birmingham Heartlands Hospital.

## Data Availability

The data that support the findings of this study are available from the corresponding author, BMF, upon reasonable request.

## Abbreviations

NIV: Noninvasive ventilation
ICU: Intensive care unit
COPD: Chronic obstructive pulmonary disease
AECOPD: Acute exacerbations of COPD
AHRF: Acute hypercapnic respiratory failure
IQR: Interquartile range
NCEPOD: National Confidential Enquiry into Patient Outcomes and Deaths.

## Supplementary Materials

Supplementary Materials Supplementary Tables 1 and 2 are tables that describe the baseline characteristics of obesity-related AHRF and non-obesity-related AHRF based on survival to discharge and in-hospital mortality rates.
