# Corrigendum to “Ward-Based Noninvasive Ventilation for Acute Hypercapnic Respiratory Failure Unrelated to Chronic Obstructive Pulmonary Disease”

**DOI:** 10.1155/carj/9846527

**Published:** 2025-06-03

B. M. Faqihi, D. Parekh, S. P. Trethewey, J. Morlet, R. Mukherjee, and A. M. Turner, “Ward-Based Noninvasive Ventilation for Acute Hypercapnic Respiratory Failure Unrelated to Chronic Obstructive Pulmonary Disease,” *Canadian Respiratory Journal* 2021, no. 1 (2021): 1–7, https://doi.org/10.1155/2021/4835536.

In the article titled “Ward-Based Noninvasive Ventilation for Acute Hypercapnic Respiratory Failure Unrelated to Chronic Obstructive Pulmonary Disease,” the following individuals were omitted from the author list:

• Syed Huq, University Hospitals Birmingham NHS Foundation Trust, Birmingham, UK
• Shyam Madathil, University Hospitals Birmingham NHS Foundation Trust, Birmingham, UK
• Kay Filby, University Hospitals Birmingham NHS Foundation Trust, Birmingham, UK

The author list should be corrected to include the above individuals due to their contributions of providing/generating data and input to the manuscript during the initial drafting.

All authors agree to this correction and agree that the abovementioned individuals meet the authorship criteria for this article.

We apologize for this error.

