# Supplementary material for: Ward-Based Noninvasive Ventilation for Acute Hypercapnic Respiratory Failure Unrelated to Chronic Obstructive Pulmonary Disease
Source: Can Respir J. 2021 Dec 21;2021:4835536. doi: 10.1155/2021/4835536 (PMC8769869; doi:10.1155/2021/4835536)
Supplement: Supplementary Materials — Supplementary Tables 1 and 2 are tables that describe the baseline characteristics of obesity-related AHRF and non-obesity-related AHRF based on survival to discharge and in-hospital mortality rates. [file 4835536.f1.docx]

Supplementary File

| Supplementary Table 1. Participant baseline characteristics (Obesity-related AHRF) | | | | |
| --- | --- | --- | --- | --- |
|  | **Median [IQR] or *n (%)*** | | |  |
|  | Total | Survival to discharge | In-hospital mortality |  |
| Characteristic | n = 188 | n = 175 | n = 13 | P value |
| Age (years) | 69 [60-75] | 69 [60-75] | 72 [66-77] | 0.214 |
| Male | *66 (35.1)* | *63 (95.5)* | *3 (4.5)* | <0.001 |
| Female | *122 (64.9)* | *112 (91.8)* | *10 (8.2)* | <0.001 |
| Pre-NIV pH | 7.27 [7.23-7.31] | 7.27 [7.24-7.31] | 7.17 [7.14-7.27] | <0.001 |
| *Pre-NIV pH groups* |  |  |  |  |
| pH > 7.15 | 16 (8.5) | 12 (75.0) | 4 (25.0) | 0.046 |
| pH 7.15 – 7.25 | 50 (26.6) | 46 (92.0) | 4 (8.0) | <0.001 |
| pH < 7.25 | 122 (64.9) | 117 (95.9) | 5 (4.1) | <0.001 |
| NIV failure | 15 (8.0) | 4 (26.7) | 11 (73.3) | 0.071 |
| Subgroup (Hospital A) |  |  |  |  |
| Duration of NIV (days) | 6 [4-10] | 6 [4-10] | 5 [3.5-5] | 0.042 |
| RF to NIV (minutes) | 123 [63.5 – 302.5] | 123 [69.0 – 288.5] | 145 [69.0 – 277.5] | 0.539 |
| Domiciliary NIV | 27 (14.4) | 27 (100.0) | 0 (0.0) | <0.001 |
| Abbreviations: IQR, inter‐quartile range; NIV, non-invasive ventilation; RF, respiratory failure, AHRF: acute hypercapnic respiratory failure. | | | | |

| Supplementary Table 2. Participant baseline characteristics (non-obesity related AHRF) | | | | |
| --- | --- | --- | --- | --- |
|  | **Median [IQR] or *n (%)*** | | |  |
|  | Total | Survival to discharge | In-hospital mortality |  |
| Characteristic | n = 291 | n = 209 | n = 82 | P value |
| Age (years) | 76 [65.75-84] | 74.5 [61-83] | 81 [70.75-87] | <0.001 |
| Male | *126 (43.3)* | *89 (70.6)* | *37 (29.4)* | <0.001 |
| Female | *165 (56.7)* | *120 (72.7)* | *45 (27.3)* | <0.001 |
| *Condition* |  |  |  |  |
| Pneumonia | *53 (18.2)* | *36 (67.9)* | *17 (32.1)* | 0.009 |
| Bronchiectasis | *40 (13.7)* | *30 (75.0)* | *10 (25.0)* | 0.002 |
| NMD/MSK | *85 (29.2)* | *63 (74.1)* | *22 (25.9)* | <0.001 |
| Fluid Overload | *48 (16.5)* | *35 (72.9)* | *13 (27.1)* | 0.001 |
| Other | *65 (22.3)* | *45 (69.2)* | *20 (30.8)* | 0.002 |
| Pre-NIV pH | 7.26 [7.20-7.31] | 7.27 [7.21-7.31] | 7.23 [7.17-7.29] | 0.007 |
| *Pre-NIV pH groups* |  |  |  |  |
| pH > 7.15 | 45 (15.5) | 30 (66.7) | 15 (33.3) | 0.025 |
| pH 7.15 – 7.25 | 88 (30.2) | 57 (64.8) | 31 (35.2) | 0.006 |
| pH < 7.25 | 158 (54.3) | 122 (77.2) | 36 (22.8) | <0.001 |
| NIV failure | 86 (29.6) | 18 (20.9) | 68 (79.1) | <0.001 |
| Subgroup (Hospital A) |  |  |  |  |
| Duration of NIV (days) | 4 [2-9] | 7 [4-10] | 5 [3.5-5] | 0.031 |
| RF to NIV (minutes) | 122 [60.0 – 316.0] | 120 [60.0 – 321.0] | 136.5 [73.5 – 297.75] | 0.476 |
| Domiciliary NIV | 17 (11.1) | 16 (94.1) | 1 (5.9) | <0.001 |
| Abbreviations: IQR, inter‐quartile range; NIV, non-invasive ventilation; RF, respiratory failure, AHRF: acute hypercapnic respiratory failure. | | | | |
